# Simultaneous Infection With Genetically Distinct *Trypanosoma cruzi* Isolates Is Associated With Distinct Myocardial Innate Immune Transcriptional Profiles During Acute Infection in Mice

**DOI:** 10.1111/pim.70103

**Published:** 2026-10-07

**Authors:** George Harisson Felinto Sampaio, Andressa Noronha Barbosa‐Silva, Carlos Ramon do Nascimento Brito, Lucas Abrantes Batista, Christiane Carlos Araújo de Negreiros, Ramayana Morais de Medeiros Brito, Rand Randall Martins, Manuela Sales Lima Nascimento, Antonia Claudia Jácome da Câmara, Paulo Marcos da Matta Guedes, Lúcia Maria da Cunha Galvão

**Affiliations:** ^1^ Programa de Pós‐Graduação em Ciências da Saúde, Centro de Ciências da Saúde Universidade Federal do Rio Grande do Norte (UFRN) Natal Rio Grande do Norte Brazil; ^2^ Programa de Pós‐Graduação em Biologia Parasitária Centro de Biociências, UFRN Natal Rio Grande do Norte Brazil; ^3^ Departamento de Análises Clinicas e Toxicológicas Centro de Ciências da Saúde, UFRN Natal Rio Grande do Norte Brazil; ^4^ Programa de Pós‐Graduação em Ciências Farmacêuticas CCS/UFRN Natal Rio Grande do Norte Brazil; ^5^ Division of Biomedical Science and Biochemistry, Research School of Biology The Australian National University Canberra Australia; ^6^ Departamento de Microbiologia e Parasitologia Centro de Biociências, UFRN Natal Rio Grande do Norte Brazil

**Keywords:** immune signalling, innate immunity, NOD‐like receptors, simultaneous infection, toll‐like receptors, *Trypanosoma cruzi*

## Abstract

Innate immune receptors are central to host resistance against *Trypanosoma cruzi* infection, yet how parasite genetic diversity is associated with these responses remains poorly understood. Here, we characterized acute infection outcomes and myocardial innate immune transcriptional profiles in mice subjected to single and mixed infections with genetically distinct *T. cruzi* parasites (DTUs TcII, TcIII, and TcVI). Swiss mice were infected with RN2, RN23, Y, or CL‐Brener individually or in combination, and parasitemia, survival, and cardiac gene expression were assessed. The Y strain, CL‐Brener clone, and RN2 + RN23 + CL‐Brener condition exhibited a high‐severity profile, with marked parasitemia (8000–28,000 trypomastigotes/5 μL), mortality of 74%–100%, reduced myocardial transcription of *Tlr2*, *Tlr4*, *Tlr9*, *Trif*, and *Myd88*, and increased transcription of *Nlrp3*, *Casp1*, *Il1β*, and *Tnfα*. In contrast, RN2 and RN23 single infections exhibited low‐severity profiles and higher transcription of innate immune mediators associated with parasite control. Mixed‐infection groups displayed transcriptional and infection profiles differing from the corresponding single‐infection groups under a standardized total inoculum. Together, these findings identify distinct myocardial innate immune transcriptional profiles across single and mixed *T. cruzi* infection conditions and support further investigation of the contributions of parasite population composition and dose to acute infection outcomes.

## 1 Introduction

*Trypanosoma cruzi* exhibits extensive genetic variability and is classified into seven *Discrete Typing Units (DTUs) (TcI–TcVI* and *TcBat)*, which differ in virulence, tissue tropism, and clinical impact, although all can infect humans and cause the diverse clinical manifestations of Chagas disease [1, 2, 3, 4]. In endemic regions, individuals may acquire multiple DTUs through triatomine vectors carrying mixed parasite populations or through repeated reinfection, increasing intra‐host genetic diversity and potentially contributing to heterogeneous infection profiles [5, 6]. Mixed infections have been widely documented in both vertebrate hosts and triatomines [7, 8] and are likely to modulate immune responses and influence Chagas disease pathogenesis [9, 10, 11]. Previous experimental and clinical studies have suggested that mixed *T. cruzi* infections and parasite population complexity may be associated with changes in parasite population dynamics, tissue distribution, parasitemia, clinical heterogeneity, and Chagas disease progression [7, 8, 9, 10, 11, 12, 13]. These findings support the idea that genetically diverse parasite populations may shape host–parasite interactions beyond the effects of individual strains alone. In experimental mouse models, the initial immune response to *T. cruzi* is triggered by the recognition of parasite‐associated molecular patterns through host pattern recognition receptors (PRRs) [14, 15]. Toll‐like receptors (TLRs) and NOD‐like receptors (NLRs) are major PRRs that regulate host defense against *T. cruzi* [16, 17, 18]. Several parasite molecules act as TLR agonists, including glycosylphosphatidylinositol (GPI), which activates TLR2 [19]. NLRs also contribute to host responses against protozoan parasites, including *T. cruzi* [20]. Glycoinositolphospholipid (GIPL) triggers NF‐κB via TLR4 [21], whereas parasite nucleic acids activate endosomal TLRs, with DNA recognized through TLR9 and RNA through TLR7 [22, 23]. To date, in contrast to TLRs, the *T. cruzi* molecules capable of activating NLRs remain unidentified. Upon recognition, macrophages internalize the parasite and release interleukin‐12 (IL‐12), which activates natural killer (NK) cells and promotes interferon‐gamma (IFN‐γ) production [24]. In conjunction with tumour necrosis factor‐alpha (TNF‐α), IFN‐γ induces inducible nitric oxide synthase (iNOS) expression, resulting in nitric oxide (NO) production that limits intracellular parasite growth [25]. Conversely, IL‐10 and transforming growth factor‐beta (TGF‐β) downregulate iNOS, dampening inflammation while favouring parasite persistence [26]. Recent evidence shows that highly virulent single infections with *T. cruzi* in mice suppress the transcription of key innate immune receptors, adaptor molecules, and cytokines essential for resistance to infection, including *Tlr2*, *Tlr4*, *Tlr9*, *Trif*, *Myd88*, and *Il12*. Conversely, these strains strongly upregulate *Nlrp3*, *Casp1*, *Il1β*, *Tnfα*, and *Inos*, which are linked to inflammatory tissue damage [27]. However, whether mixed infection conditions involving genetically distinct *T. cruzi* populations with different virulence profiles are associated with acute infection outcomes and myocardial innate immune transcriptional patterns that differ from those observed in single infections remains unclear. In this study, we characterized the course of infection and myocardial innate immune gene transcription under single and mixed *T. cruzi* infection conditions during the acute phase in mice, with particular emphasis on TLRs, NLRs, adaptor molecules, and cytokine expression.

## 2 Materials and Methods

### 2.1 Ethics Statement

All experimental procedures involving Swiss mice were conducted in accordance with the guidelines of the Ethics Committee on Animal Use (CEUA) of the Federal University of Rio Grande do Norte (UFRN) and were approved under protocol no. 066/2018.

### 2.2 *Trypanosoma cruzi* Used for Experimental Infections

The *T. cruzi* parasites used in this study included RN23 (DTU II), obtained from a human patient residing in Serra Negra do Norte, Rio Grande do Norte, Brazil, and RN2 (DTU III), isolated from a naturally infected *Triatoma brasiliensis* collected indoors in the same municipality [28]. In addition, the Y strain (DTU II), originally isolated from a patient in the acute phase of Chagas disease [29], and the CL‐Brener clone (DTU VI) [30], derived from the CL strain isolated from a naturally infected *Triatoma infestans* in Rio Grande do Sul [31], were included. All *T. cruzi* parasites were cryopreserved in liquid nitrogen at −196°C in the Laboratory of Parasite Biology and Chagas Disease (LABIOPAR), Department of Clinical and Toxicological Analyses, Health Sciences Center, UFRN. Before use in the experiments, the parasites were thawed and maintained through five successive passages in male Swiss mice, as previously established [32].

### 2.3 Experimental Infection of Mice

Male Swiss outbred mice (4–6 weeks old, 18–24 g) were randomly allocated into seven experimental groups: Group 1, non‐infected controls; Group 2, mice inoculated with RN2 (DTU III); Group 3, mice inoculated with RN23 (DTU II); Group 4, mice inoculated with CL‐Brener (DTU VI); Group 5, mice inoculated with the Y strain (DTU II); Group 6, mice inoculated with RN2 + RN23; and Group 7, mice inoculated with RN2 + RN23 + CL‐Brener. For parasitemia and survival analyses, seven mice per group were included in each experiment, and the entire experimental design was performed twice independently, resulting in 14 mice analysed per group. For myocardial gene expression analysis, cardiac tissue samples from three randomly selected mice per experimental group in each independent experiment were collected at 15 days post‐infection and analysed by qPCR, resulting in a total of six mice per group. This balanced sampling strategy was adopted because the high mortality observed under high‐severity infection conditions during the acute phase limited the number of cardiac tissue samples available at the collection time point. The same qPCR dataset was used for principal component analysis and heatmap generation. Thus, parasitemia and survival analyses were performed with *n* = 14 mice per group, whereas gene expression, PCA, and heatmap analyses were performed with *n* = 6 mice per group. Mice were randomly assigned to the experimental groups, and investigators responsible for parasitemia counting and molecular analyses were blinded to group allocation whenever possible. Infections were performed by intraperitoneal inoculation with a total of 1 × 10^4^ bloodstream trypomastigotes per mouse. For mixed infections, the total inoculum was maintained at 1 × 10^4^ bloodstream trypomastigotes and divided equally among the parasite populations included in each group. Therefore, in the RN2 + RN23 + CL‐Brener group, each parasite population contributed approximately 3.3 × 10^3^ parasites, including CL‐Brener, whereas the CL‐Brener single‐infection group received 1 × 10^4^ CL‐Brener parasites.

### 2.4 Parasitemia and Survival

Parasitemia was monitored daily from Day 3 to Day 30 post‐infection, following the method described by Pizzi [33] and modified by Brener [32]. Approximately 5 μL of peripheral blood was collected from the tail vein of each mouse and examined under a coverslip (22 × 22 mm) by light microscopy at ×400 magnification. Bloodstream trypomastigotes were counted in 50 random microscopic fields. Animal survival was monitored daily for up to 90 days post‐infection. Survival curves were constructed using the Kaplan–Meier method, and differences between groups were analysed using the log‐rank (Mantel–Cox) test. Mice were also observed daily for clinical signs of infection, including reduced mobility, piloerection, weight loss, and lethargy. Animals showing severe clinical deterioration were euthanized according to the humane endpoints established in the experimental protocol.

### 2.5 Quantification of Innate Immune Receptor, Adaptor Molecule, Cytokine, and *Inos* mRNA Expression in Cardiac Tissue

At 15 days post‐infection, corresponding to the acute phase of experimental *T. cruzi* infection, the mice were anaesthetised and euthanized by intraperitoneal administration of an overdose of ketamine (150 mg/kg) combined with xylazine (30 mg/kg). The thoracic cavity was opened and heart tissue fragments were collected. Approximately 25–30 mg of cardiac tissue from each animal were immediately preserved in tubes containing 500 μL of TRIzol reagent (Invitrogen, Carlsbad, CA, USA). Total RNA was extracted using TRIzol and the SV Total RNA Isolation System (Promega, Madison, WI, USA), including DNase treatment, following the manufacturers' instructions. RNA samples were stored at −80°C, and concentration and purity were assessed using a NanoDrop 2000 spectrophotometer (Thermo Scientific, Waltham, MA, USA). Complementary DNA (cDNA) was synthesized from 2 μg of total RNA using the High‐Capacity cDNA Reverse Transcription Kit (Applied Biosystems, USA). Quantitative PCR (qPCR) assays were performed using Fast SYBR Green Master Mix (Applied Biosystems, USA) and gene‐specific primers (Table 1). All qPCR reactions were performed in triplicate, and mean Ct values were used for relative expression analysis. Amplification reactions were performed using a 7500 Fast Real‐Time PCR System (Applied Biosystems) in 96‐well MicroAmp plates under the following conditions: initial denaturation at 50°C for 2 min and 95°C for 10 min, followed by 40 cycles of 94°C for 30 s, annealing at gene‐specific temperatures for 30 s, and 72°C for 1 min. Gene transcription levels of innate immune receptors (*Tlr2*, *Tlr4*, *Tlr9*, and *Nlrp3*), adaptor molecules (*Myd88*, *Trif*, and *Casp1*), cytokines (*Il1β*, *Il6*, *Il10*, *Il12p35*, *Il12p40*, *Ifnγ*, and *Tnfα*), and *Inos* were first normalized to the housekeeping gene glyceraldehyde‐3‐phosphate dehydrogenase (*Gapdh*) and then expressed relative to the expression levels observed in uninfected control cardiac tissue, which was used as the calibrator group, using the 2^*−*ΔΔCt^ method.

**TABLE 1:** Sequences of the gene‐specific primers used in the real‐time quantitative PCR assays.

Locus	Sense (5′‐3′)	Antisense (5′‐3′)
*Gapdh*	TGCAGTGGCAAAGTGGAGAT	CGTGAGTGGAGTCATACTGGAA
*Tlr2*	CGAGTGGTGCAAGTACG	GGTAGGTCTTGGTGTTCATTATC
*Tlr4*	CCTCTGCCTTCACTACAGAGACTTT	TGTGGAAGCCTTCCTGGATG
*Tlr9*	CTGCCGCTGACTAATCTG	CTGAAATTGTGGCCTATACCC
*Myd88*	TGATGCGGAGCCAGATT	GAGGAGGCATGTGTGTACT
*Trif*	ATTTCAGGTGCCCGGGCGTG	TTTGCCGCTCTGCCTCCAGC
*Casp1*	AGATGGCACATTTCCAGGAC	CCTCCAGCAGCAACTTC
*Il1β*	GCAACTGTTCCTGAACTCAACT	ATCTTTTGGGGTCCGTCAACT
*Il6*	CCATCCAGTTGCCTTCTTG	AAGTGCATCATCGTTGTTCATAC
*Il10*	TGGACAACATACTGCTAACC	GGATCATTTCCGATAAGGCT
*Il12p35*	TCTCTGGACCTGCCAGGTGT	CCTGTTGATGGTCACGACGCG
*Il12p40*	CAACATCAAGAGCAGTAGCAG	TACTCCCAGCTGACCTCCAC
*Tnfα*	TGTGCTCAGAGCTTTCAACAA	CTTGATGGTGGTGCATGAGA
*Ifnγ*	GCATCTTGGCTTTGCAGCT	CCTTTTTCGCCTTGCTGTTG
*Inos*	CGAAACGCTTCACTTCCAA	TGAGCCTATATTGCTGTGGCT

### 2.6 Statistical Analysis

Statistical analyses were performed using Stata version 15.0 (StataCorp, College Station, TX, USA). qPCR data were normalized to the housekeeping gene *Gapdh* and expressed as relative expression values using the uninfected control group as the calibrator, according to the 2^−ΔΔCt^ method. These normalized relative expression values were used for group comparisons, principal component analysis, and heatmap generation. Data are expressed as mean ± standard error of the mean (SEM). The Shapiro–Wilk test was used to assess normality, and the results for gene expression variables are provided in TableS1. Normally distributed data were analysed using one‐way analysis of variance (ANOVA) followed by Bonferroni's post hoc test. Non‐parametric data were analysed using the Kruskal–Wallis test followed by Dunn's post hoc test. Survival curves were analysed using the Kaplan–Meier method and compared using the log‐rank (Mantel–Cox) test. A *p* ≤ 0.05 was considered statistically significant. Principal component analysis and heatmap analyses were performed using the ClustVis online tool [34]. Briefly, these normalized relative expression values were organized as a matrix containing the measured parameters in rows and the experimental groups in columns. PCA was performed using singular value decomposition, and the first two principal components were plotted to visualize the distribution and clustering of the experimental groups based on their normalized gene expression profiles. Heatmaps were generated from the standardized dataset using correlation‐based distance and average linkage clustering to identify similarities among the measured parameters and experimental groups.

## 3 Results

### 3.1 Susceptibility of Mice to Single or Mixed Infections With Different *T. cruzi* Parasite Populations

The assessment of parasitemia and survival/mortality in mice infected with distinct *T. cruzi* parasites from different biological sources, including two field isolates (RN2 and RN23), the Y reference strain, and the CL‐Brener clone, as well as in animals subjected to mixed infections (RN2 + RN23 or RN2 + RN23 + CL‐Brener), revealed two clearly distinct infection severity profiles: high and low. The infection conditions involving the Y strain, the CL‐Brener clone, and RN2 + RN23 + CL‐Brener were characterized by a high‐severity profile. Mice infected under these conditions exhibited peak parasitemias ranging from 8000 to 28,000 trypomastigotes per 5 μL of blood and mortality rates between 74% and 100% (Figure 1A,B). In contrast, single infections with either RN2 or RN23, as well as the RN2 + RN23 mixed infection, exhibited a markedly less severe infection profile, with peak parasitemias below 4000 trypomastigotes per 5 μL of blood and 0% mortality (Figure 1A,B). Peak parasitemias in mice infected with RN2, RN23, and RN2 + RN23 were 864, 253, and 3123 trypomastigotes per 5 μL of blood, respectively. Interestingly, mice with the RN2 + RN23 mixed infection showed higher peak parasitemias than those observed in the corresponding single‐infection groups. Conversely, mixed infection with three parasite populations (RN2 + RN23 + CL‐Brener) also showed a profile distinct from the corresponding single infections with RN2, RN23, or CL‐Brener. In this case, mice infected with RN2 + RN23 + CL‐Brener displayed lower parasitemia and lower mortality than those infected with the CL‐Brener clone alone (Figure 1A,B). Under the fixed total inoculum used in this study, RN2 + RN23 showed higher parasitemia than the corresponding single infections, whereas RN2 + RN23 + CL‐Brener showed lower parasitemia and mortality than CL‐Brener alone.

**FIGURE 1:**
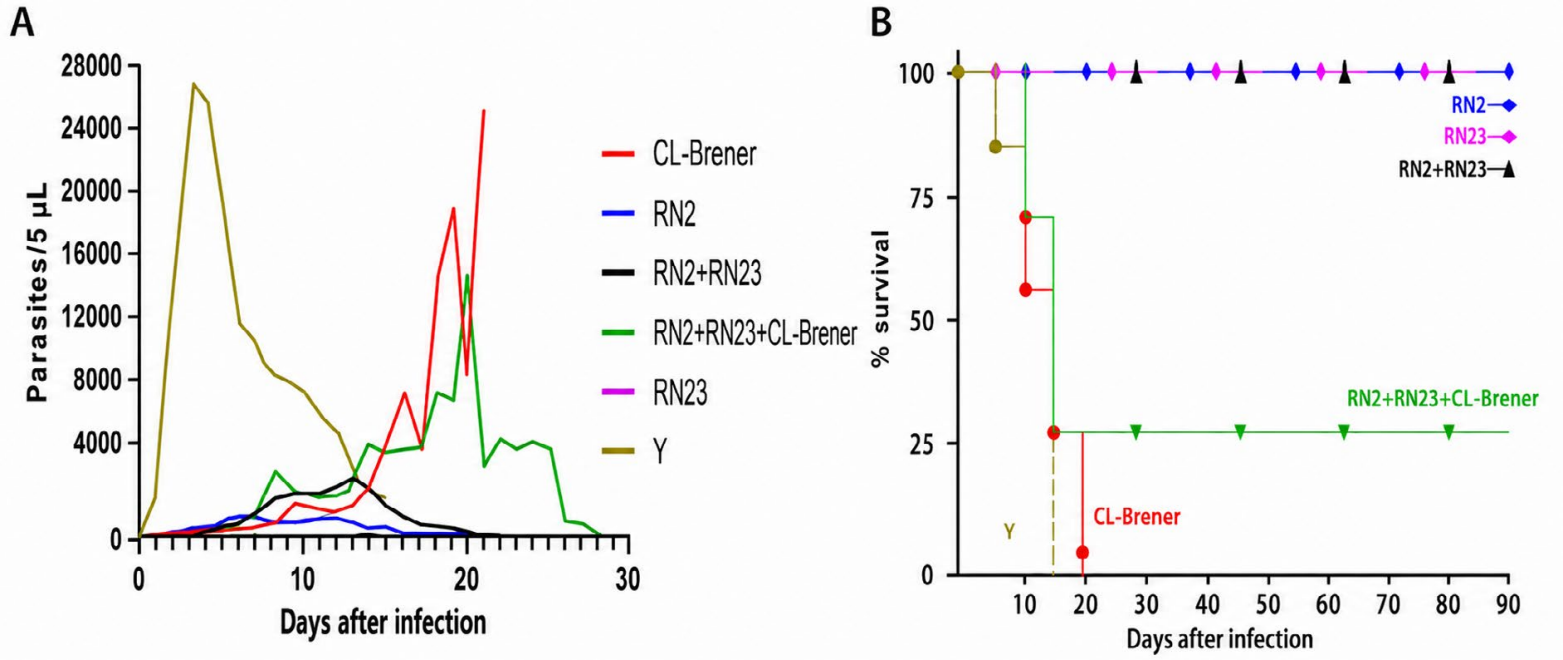
Experimental infection of Swiss mice with *Trypanosoma cruzi* parasites from different DTUs (A) Parasitemia in mice intraperitoneally infected with 1 × 10^4^ bloodstream trypomastigotes. (B) Survival curves of infected mice. Data represent the combined results of two independent experiments. Survival curves were compared using the log‐rank (Mantel–Cox) test. *n* = 14 mice per group.

### 3.2 Differential Myocardial Transcription of TLR and NLR Genes in Mice Subjected to Single or Mixed *Trypanosoma cruzi* Infections

The transcription of genes encoding TLRs and NLRs in the cardiac tissue of mice infected with different *T. cruzi* parasite populations, under both single and mixed infections, was quantitatively evaluated to determine whether distinct infection profiles were associated with differential transcription of innate immune receptors and adaptor molecules. Mice singly infected with high‐virulence parasites (Y strain or CL‐Brener clone) exhibited significantly lower transcription levels of *Tlr2*, *Tlr4*, *Myd88*, and *Trif* than mice with single infections caused by low‐virulence parasites (RN2 or RN23) (Figure 2A–E). Thus, single infections with highly virulent parasites showed reduced myocardial transcription of genes encoding key components of innate immune signalling pathways involved in early parasite recognition and host resistance. Conversely, mixed infection with two low‐virulence parasites (RN2 + RN23) was associated with a distinct pattern of innate immune receptor transcription and higher parasitemia than that observed in the corresponding single‐infection groups. Mice simultaneously infected with RN2 + RN23 showed reduced transcription levels of *Tlr2* and *Tlr4*, while *Tlr9*, *Myd88*, and *Trif* levels were not significantly different from those observed in mice singly infected with RN2 or RN23 (Figure 2A–E).

**FIGURE 2:**
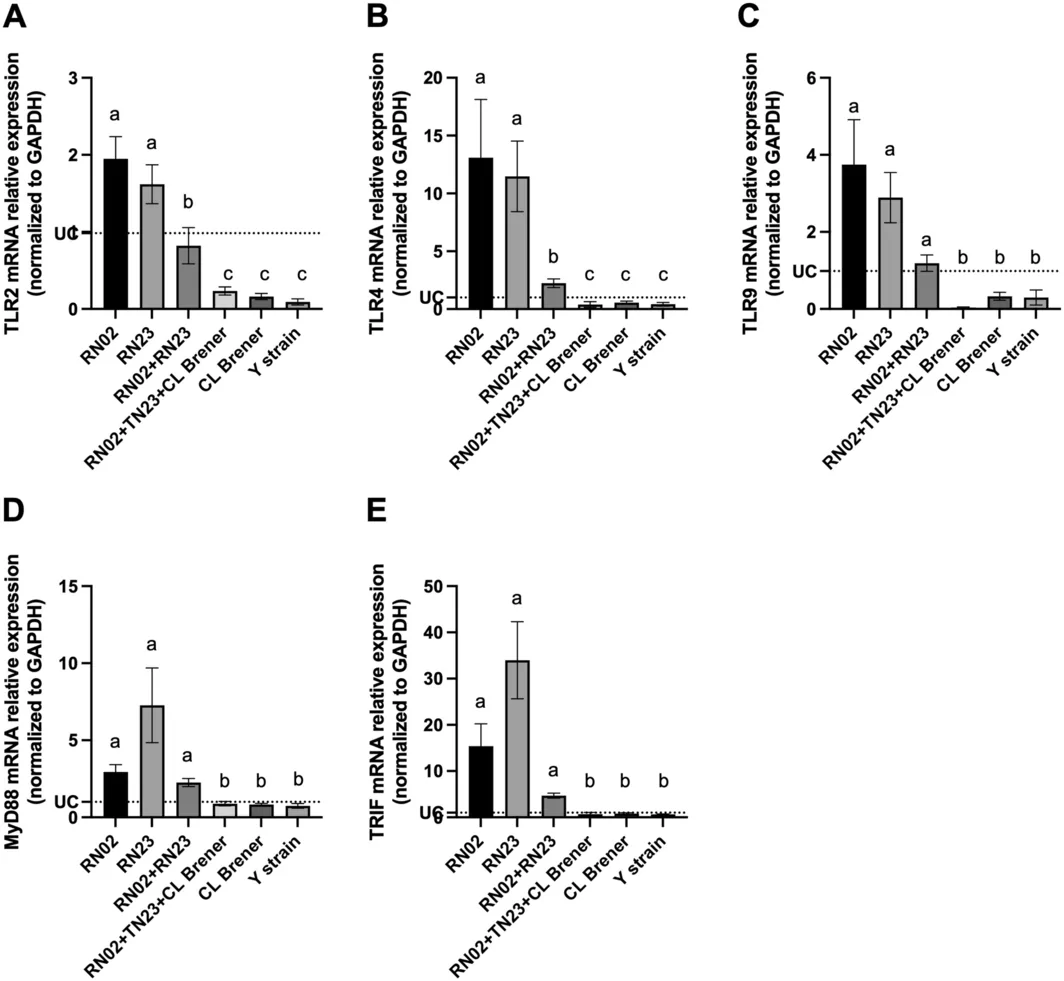
Reduced myocardial transcription of *Tlr2*, *Tlr4*, *Tlr9*, *Myd88*, and *Trif* across *Trypanosoma cruzi* infection conditions during the acute phase. Relative mRNA expression of *Tlr2* (A), *Tlr4* (B), *Tlr9* (C), *Myd88* (D), and *Trif* (E) was determined by real‐time quantitative PCR in heart tissue from Swiss mice infected intraperitoneally with 1 × 10^4^ *T. cruzi* bloodstream trypomastigotes. Expression levels were normalized to the housekeeping gene *Gapdh* and expressed relative to the uninfected control group (UC) using the 2^−ΔΔCt^ method. The dotted line in each graph represents the mean relative mRNA expression value of UC, corresponding to the reference value of 1.0 in the 2^−ΔΔCt^ analysis. Data are presented as mean ± standard error. Group comparisons were performed using the Kruskal–Wallis test followed by Dunn's multiple‐comparison post hoc test, based on the results of the Shapiro–Wilk normality test Table(S1). Different letters indicate statistically significant differences among groups (*p* ≤ 0.05). *n* = 6 mice per group.

### 3.3 Reduced Myocardial Transcription of *Il6* , *Il10* , *Il12p35* , and *Il12p40* in High‐Severity *T. cruzi* Infection Conditions

High‐severity infection conditions (Y, CL‐Brener, or RN2 + RN23 + CL‐Brener) were associated with reduced transcription of *Il10*, *Il12p35*, and *Il12p40* in the myocardium, showing statistically significant differences when compared with lower‐severity infection conditions (RN2, RN23, or RN2 + RN23) (Figure 3A–C). Notably, *Il10*, *Il12p40*, and *Il12p35* mRNA levels were lowest in the three‐parasite combination (RN2 + RN23 + CL‐Brener), differing significantly from infections with RN2, RN23, or RN2 + RN23 (Figure 3A–C). The greatest reduction in *Il6* mRNA expression was observed in mice infected with the Y strain compared to those infected with the RN2 isolate (Figure 3D). Regardless of infection severity profile or infection type (single or mixed), mice exhibited similar *Ifnγ* transcription levels in the cardiac tissue, with no statistically significant differences detected (Figure 3E).

**FIGURE 3:**
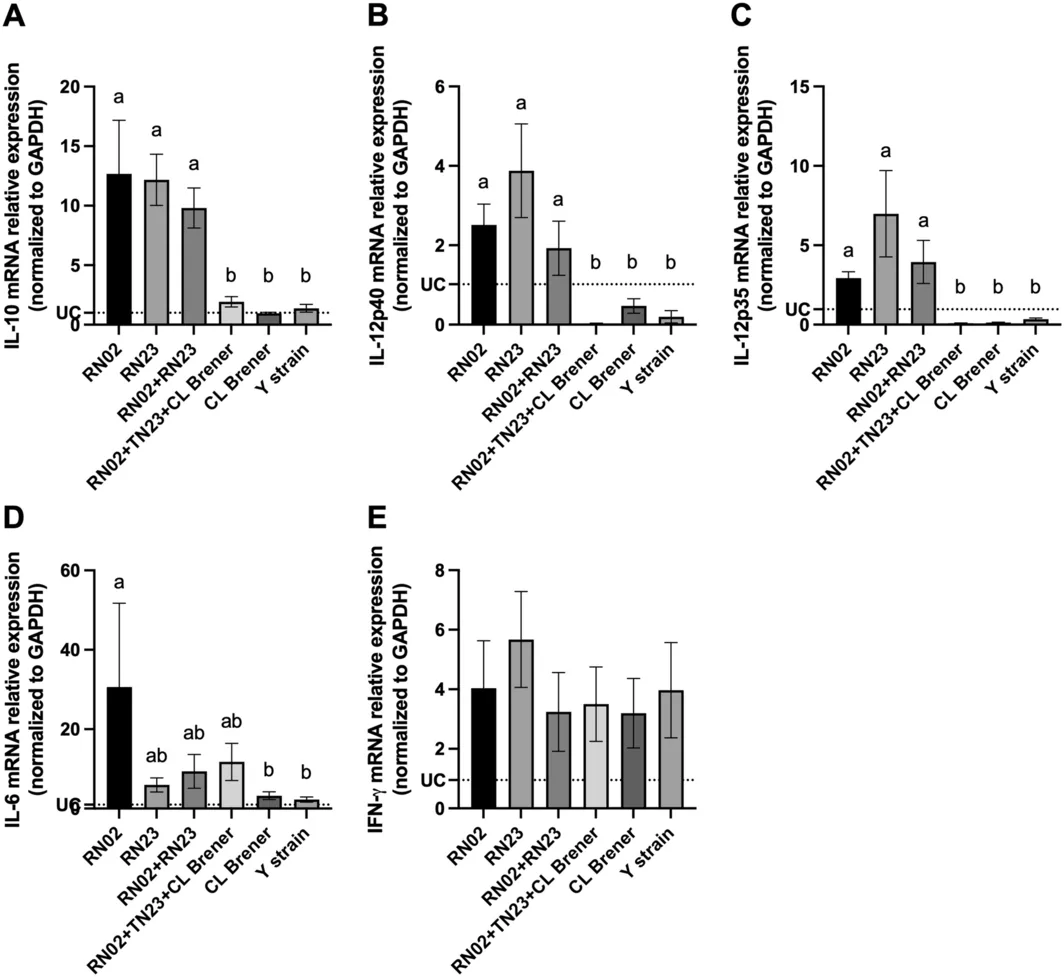
Differential myocardial cytokine gene transcription under single and mixed *Trypanosoma cruzi* infection conditions during the acute phase. Relative mRNA expression of *Il10* (A), *Il12p40* (B), *Il12p35* (C), *Il6* (D), and *Ifnγ* (E) was determined by real‐time quantitative PCR in heart tissue from Swiss mice intraperitoneally infected with 1 × 10^4^ *T. cruzi* bloodstream trypomastigotes. Gene expression was normalized to the housekeeping gene *Gapdh* and expressed relative to the uninfected control group (UC) using the 2^−ΔΔCt^ method. The dotted line in each graph represents the mean relative mRNA expression value of UC, corresponding to the reference value of 1.0 in the 2^−ΔΔCt^ analysis. Data are presented as mean ± standard error. Group comparisons were performed using the Kruskal–Wallis test followed by Dunn's multiple‐comparison post hoc test, based on the results of the Shapiro–Wilk normality test Table(S1). Different letters indicate statistically significant differences among groups (*p* ≤ 0.05). *n* = 6 mice per group.

### 3.4 High‐Severity *T. cruzi* Infection Conditions Are Associated With Increased Transcription of *Nlrp3*, *Il1β*, and *Tnfα*

High‐severity infection conditions (Y, CL‐Brener, or RN2 + RN23 + CL‐Brener) were associated with increased transcription of *Nlrp3*, *Il1β*, and *Tnfα*, showing statistically significant differences compared with lower‐severity infection conditions (RN2, RN23, or RN2 + RN23) (Figure 4A–D). Mice infected with the Y strain, which exhibited the highest parasitemia, also showed the highest *Casp1* mRNA expression relative to all other infected groups (Figure 4B). The combined transcriptional profiles of PRRs, adaptor molecules, and cytokines differed among mice under single and mixed *T. cruzi* infection conditions, as indicated by PCA and heatmap analyses (Figure 5A–C). In this analysis, lower‐ and high‐severity infection conditions occupied partially distinct regions of the PCA space. Interestingly, mixed infection with two low‐virulence parasite populations was associated with higher parasitemia than the corresponding single infections and showed an intermediate position in the PCA space based on the combined transcriptional profiles of innate immune receptors, adaptor molecules, and cytokines, between lower‐ and high‐severity infection conditions (Figure 5A). In addition, mice infected with the three‐parasite combination RN2 + RN23 + CL‐Brener showed 74% mortality, compared with 100% mortality in mice infected with CL‐Brener alone. Although the mixed triple infection (RN2 + RN23 + CL‐Brener) clustered together with single infections involving virulent parasites, differences in the expression profiles of the immune markers evaluated were detectable. These differences, however, were not sufficient to generate a separate cluster in the PCA (Figure 5A).

**FIGURE 4:**
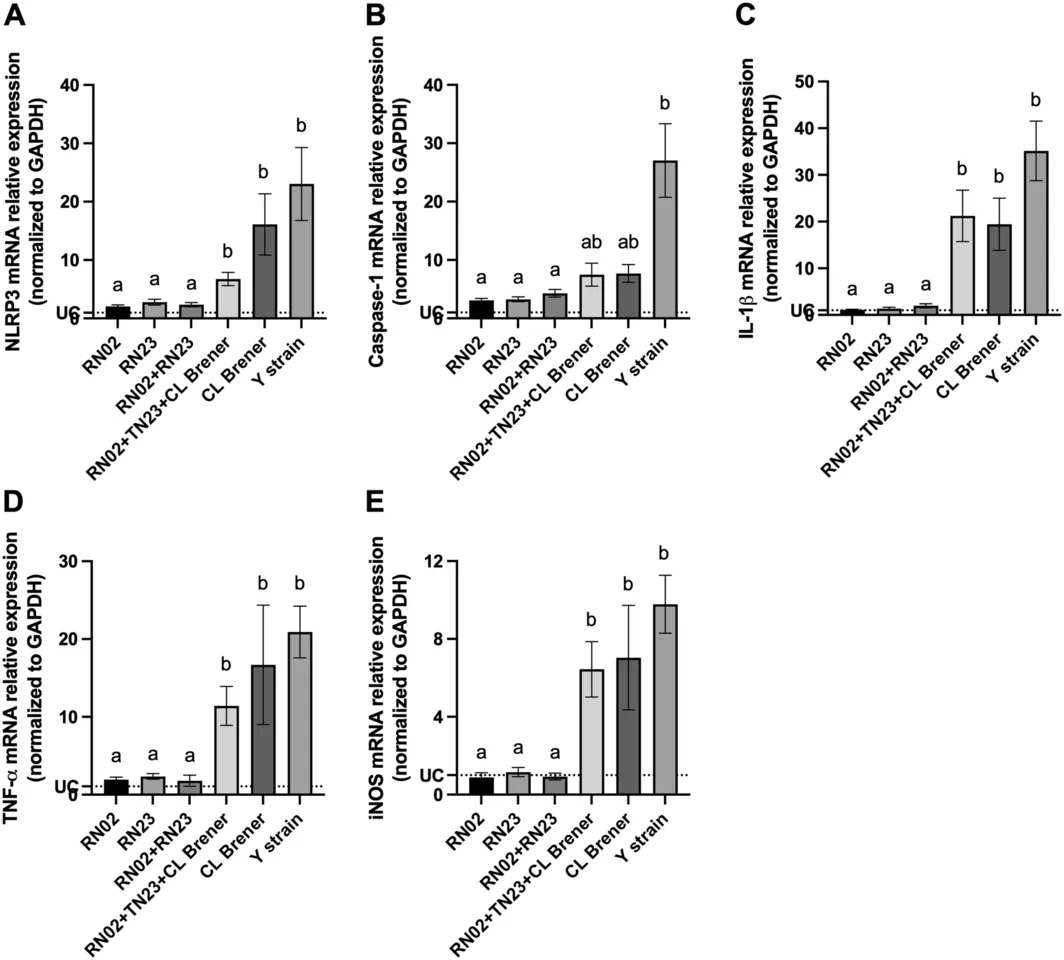
Differential myocardial transcription of inflammasome‐ and inflammation‐related genes under single and mixed *Trypanosoma cruzi* infection conditions during the acute phase. Relative mRNA expression of *Nlrp3* (A), *Casp1* (B), *Il1β* (C), *Tnfα* (D), and *Inos* (E) was determined by real‐time quantitative PCR in heart tissue from Swiss mice intraperitoneally infected with 1 × 10^4^ *T. cruzi* bloodstream trypomastigotes. Gene expression was normalized to the housekeeping gene *Gapdh* and expressed relative to the uninfected control group (UC) using the 2^−ΔΔCt^ method. The dotted line in each graph represents the mean relative mRNA expression value of UC, corresponding to the reference value of 1.0 in the 2^−ΔΔCt^ analysis. Data are presented as mean ± standard error. Group comparisons were performed using the Kruskal–Wallis test followed by Dunn's multiple‐comparison post hoc test, based on the results of the Shapiro–Wilk normality test Table (S1). Different letters indicate statistically significant differences among groups (*p* ≤ 0.05). *n* = 6 mice per group.

**FIGURE 5:**
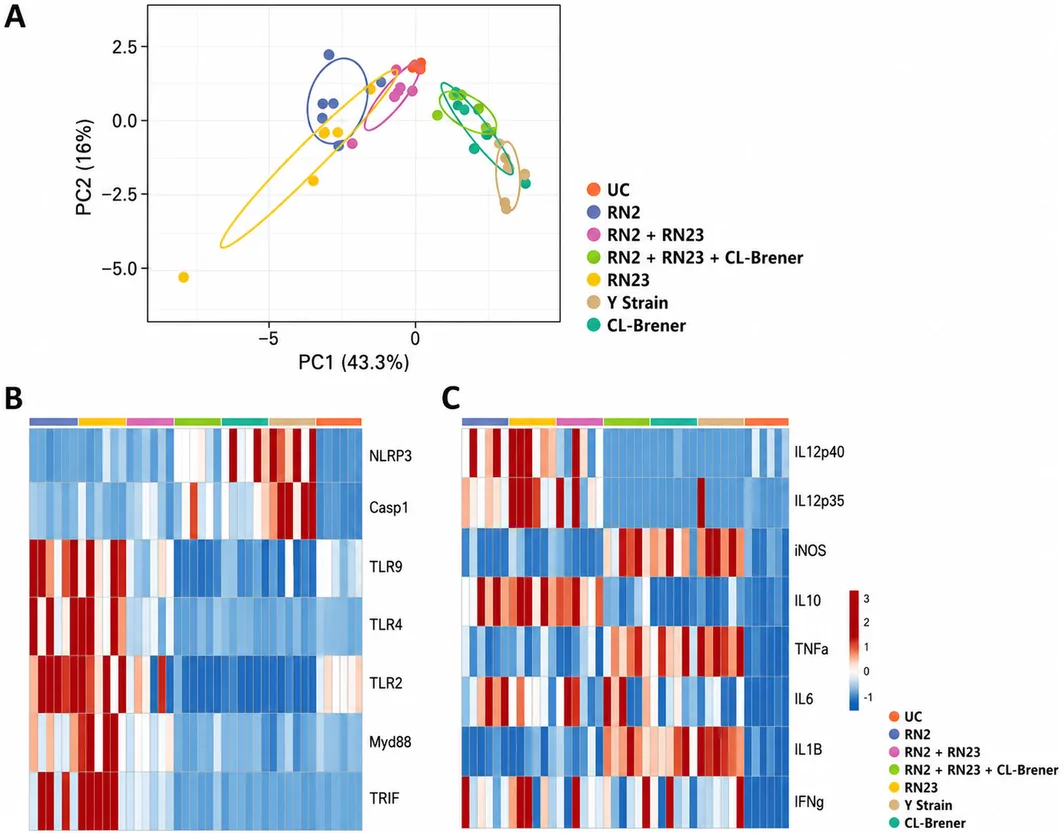
Principal component analysis and heatmap reveal distinct transcriptional patterns across *Trypanosoma cruzi* infection conditions during the acute phase. Principal component analysis (PCA) and heatmap analyses were performed using normalized qPCR expression values of the immunological markers evaluated in this study. Data were normalized to the housekeeping gene *Gapdh* and expressed relative to uninfected control cardiac tissue using the 2^−ΔΔCt^ method. (A) PCA based on the combined transcriptional levels of Toll‐like receptors (TLRs), NOD‐like receptors (NLRs), inflammasome‐related molecules, cytokines, and *Inos*, showing partial separation in the PCA space among single infections with highly virulent parasites (Y and CL‐Brener), the high‐severity RN2 + RN23 + CL‐Brener mixed‐infection condition, single infections with low‐virulence parasites (RN2 and RN23), and the lower‐severity RN2 + RN23 mixed‐infection condition. (B) Heatmap of the relative mRNA expression levels of TLRs, NLRs, and adaptor molecules. (C) Heatmap of the relative mRNA expression levels of cytokines and *Inos*. PCA was performed using singular value decomposition, and heatmaps were generated from the standardized dataset using correlation‐based distance and average linkage clustering. These analyses were exploratory and were not used for parametric or non‐parametric group comparisons. *n* = 6 mice per group.

## 4 Discussion

In the present study, highly virulent *T. cruzi* infections caused by the Y strain and CL‐Brener clone were associated with reduced transcription of key TLR‐related genes involved in parasite recognition and host resistance, including *Tlr2* and *Tlr4*, together with increased transcription of the inflammasome component *Nlrp3*. We first assessed parasitemia and survival outcomes in mice subjected to single and mixed infections. Single infections with the highly virulent Y strain and CL‐Brener clone, as well as the RN2 + RN23 + CL‐Brener mixed‐infection condition, exhibited a high‐severity infection profile, yielding elevated parasitemia and mortality rates ranging from 74% to 100%. Consistent with previous reports, infections with the Y strain and CL‐Brener clone have been associated with 100% and 81% mortality, respectively [35]. In contrast, single infections with the isolates RN2 and RN23 were characterized by markedly lower virulence. Notably, the RN2 + RN23 + CL‐Brener mixed infection showed lower parasitemia and mortality than the CL‐Brener single infection, whereas the RN2 + RN23 combination showed increased parasitemia compared with the same isolates in single infection. These comparisons, however, must be interpreted in the context of the experimental design, in which the same total inoculum was maintained across all infected groups but divided among the parasite populations in mixed infections. Consequently, the present design does not allow us to determine whether the differences observed between single and mixed infections are related to parasite population composition, the dose of the individual parasite populations, or a combination of these factors.

In this study, acute infection parameters were assessed based on parasitemia, survival/mortality, and myocardial innate immune gene expression. Cardiac parasite burden and strain‐ or DTU‐specific parasite quantification were not evaluated; therefore, we could not directly associate myocardial parasite load or the relative contribution of each parasite population with the observed gene expression profiles. Future studies incorporating cardiac parasite quantification and strain‐ or DTU‐specific molecular approaches together with dose‐matched single‐infection controls will be important to better define parasite population dynamics and myocardial innate immune transcriptional responses in single and mixed *T. cruzi* infections. Moreover, parasites belonging to the same DTU, such as the Y strain and the RN23 isolate, both classified as DTU II, displayed substantial differences in virulence and parasitemia when evaluated in single infections using the same inoculum. This finding suggests that DTU classification, although important for characterizing the genetic diversity of *T. cruzi* , should be interpreted together with strain‐ or isolate‐specific features, particularly when considering parameters such as parasitemia, virulence, and mortality. Overall, these results highlight the complexity of host–parasite interactions, indicating that parasitemia and survival/mortality outcomes may reflect the interplay of multiple factors, including parasite diversity, host immune competence, and the specific experimental infection condition [3, 11, 36]. Previous studies have shown that mixed infections with genetically distinct parasite populations can be associated with changes in parasite population dynamics, blood parasitism, and tissue distribution compared with single infections [3, 36, 37]. From a mechanistic standpoint, activation of innate immune receptors represents a critical step in the early recognition of *T. cruzi* , contributing not only to initial parasite control but also to shaping the subsequent adaptive immune response [38]. PRRs such as TLR2, TLR4, and TLR9, as well as inflammasome components including NLRP3, regulate cytokine production, immune‐cell recruitment, and inflammatory amplification during infection. Importantly, *T. cruzi* exhibits extensive genetic diversity across DTUs, including variation in multigene families encoding parasite surface molecules such as trans‐sialidases, mucins, and mucin‐associated surface proteins (MASPs). These molecules play key roles in host–parasite interactions and can influence how parasite‐derived ligands are recognized by host PRRs. For example, GPI anchors and GIPLs derived from *T. cruzi* are known to activate TLR2 and TLR4, while parasite nucleic acids can stimulate endosomal TLRs such as TLR9 [19, 20, 21, 22, 23]. Consequently, parasites belonging to different DTUs may differentially activate or suppress innate immune pathways, potentially contributing to distinct cytokine profiles and parasitemia and survival/mortality outcomes. Mechanistically, previous studies have proposed that the coexistence of genetically distinct parasite populations may modulate host–parasite interactions through competition between parasite populations, differential immune activation, or immune evasion strategies. Such interactions may alter the magnitude and balance of innate immune signalling, potentially generating immunological responses that differ from those observed during single‐parasite infections [15, 38, 39]. In the present study, highly virulent *T. cruzi* infections caused by the Y strain and CL‐Brener clone, which were associated with the highest parasitemia levels and mortality among the experimental groups, were also associated with reduced transcription of *Tlr2*, *Tlr4*, *Myd88*, and *Trif*, together with increased transcription of *Nlrp3*, *Il1β*, and *Tnfα*. Mixed‐infection groups also exhibited distinct patterns of innate immune gene expression, parasitemia, and survival/mortality; however, because parasite dose and population composition were not independently controlled, these differences cannot be attributed specifically to interactions among parasite populations. These findings are consistent with distinct patterns of innate immune gene transcription associated with high‐severity *T. cruzi* infection conditions, rather than simply with a uniformly stronger inflammatory response. However, the present experimental design does not allow us to determine the extent to which the observed transcriptional differences are attributable to parasite virulence, parasite dose, population composition, or their combination. Reduced *Tlr2* and *Tlr4* expression together with increased *Nlrp3* transcription have previously been observed in mice infected with virulent *T. cruzi* strains such as Y and Colombiana [27]. Experimental studies have also shown that impaired signalling through TLR2, TLR4, or TLR9 increases susceptibility to *T. cruzi* infection, leading to higher parasitemia and mortality [19, 21, 22, 23]. Reduced TLR signalling compromises macrophage activation, decreases NO production, and facilitates parasite proliferation [21, 22, 40, 41]. Thus, reduced signalling through TLRs and their adaptor molecules may be associated with decreased macrophage activation and protective cytokine responses, thereby favouring parasite replication during acute infection. At the same time, increased transcription of *Nlrp3*, *Il1β*, and *Tnfα* is consistent with a dysregulated inflammatory environment, in which protective innate recognition is weakened while inflammasome‐associated and pro‐inflammatory pathways remain activated [27, 42, 43]. In the present study, this transcriptional profile was observed in the groups with higher mortality during the acute phase of infection and is consistent with evidence showing that activation of the NLRP3 inflammasome contributes to host responses during early *T. cruzi* infection, and that mice deficient in NLRP3 or caspase‐1/11 display higher parasitemias and exacerbated tissue damage [44]. In addition, increased IL‐1β production has been associated with cardiac dysfunction in chronic Chagas cardiomyopathy, with higher IL‐1β expression correlating with reduced left ventricular ejection fraction [45]. In patients with the cardiac form of Chagas disease, higher mRNA expression levels of genes encoding NLRP3 and IL‐1β have also been reported compared with patients with the indeterminate form of the disease [46]. Previous studies have also shown that *T. cruzi* ‐infected macrophages release increased amounts of extracellular vesicles (EVs), which interact with TLR2 and induce NF‐κB translocation, thereby promoting the production of pro‐inflammatory cytokines such as TNF‐α and IL‐1β [47]. Furthermore, pro‐inflammatory cytokine signalling, including IL‐1β, has been associated with cardiac inflammation, structural remodelling, and functional alterations in chronic Chagas disease [48, 49, 50]. Previous studies comparing single and mixed infections have suggested that mixed infections may be associated with phenotypes that differ from those observed in single infections, including changes in parasite population dynamics, parasitemia, survival/mortality profiles, and tissue tropism when genetically distinct parasite strains coexist within the same host [51, 52]. In summary, under the experimental conditions evaluated, single and mixed *T. cruzi* infections were associated with distinct patterns of myocardial innate immune gene transcription, parasitemia, and survival/mortality during the acute phase of infection in mice. These findings should be interpreted as descriptive associations rather than evidence of a specific causal effect of parasite population composition. Mixed‐infection groups nevertheless displayed transcriptional profiles that differed from those observed in the corresponding single infections, including differences in *Tlr2*, *Tlr4*, and *Nlrp3* transcription. Our results therefore confirm previous observations in highly virulent single infections while extending these findings by showing that mixed‐infection conditions are also associated with distinct myocardial innate immune transcriptional profiles. Collectively, these findings characterize distinct acute infection and myocardial innate immune transcriptional profiles across the experimental conditions evaluated, highlighting biologically relevant differences between single and mixed infections and providing a framework for future studies to disentangle the contributions of parasite dose, population composition, and parasite–parasite interactions.

## Funding

This work was supported by CNPq/MCTI/FNDCT (22/2024) (process no. 444706/2024‐0). G.H.F.S. and A.N.B.‐S. received postdoctoral fellowships from CNPq. L.M.C.G. received a research fellowship from CNPq. L.A.B. and C.C.A.N. received doctoral and master's scholarships from CAPES.

## Conflicts of Interest

The authors declare no conflicts of interest.

## Supporting information


**Table S1:** Shapiro‐Wilk normality test results for gene expression variables used to support non‐parametric statistical analyses.

## Data Availability

The data that support the findings of this study are available from the corresponding author upon reasonable request.
