# Supplementary material for: Simultaneous Infection With Genetically Distinct Trypanosoma cruzi Isolates Is Associated With Distinct Myocardial Innate Immune Transcriptional Profiles During Acute Infection in Mice
Source: Parasite Immunol. 2026 Oct 7;48(10):e70103. doi: 10.1111/pim.70103 (PMC13643500; doi:10.1111/pim.70103)
Supplement: Supplementary file 1 [file PIM-48-e70103-s001.docx]

**Supplementary Table S1**. Shapiro–Wilk normality test results for gene expression variables used to support non-parametric statistical analyses.

| Descriptive Statistics | IL12p40 | IL12p35 | iNOS | IL10 | TNFa | IL6 | IL1B | NLRP3 | Caspase1 | TLR9 | TLR4 | TLR2 | IFNg | Myd88 | TRIF |
| --- | --- | --- | --- | --- | --- | --- | --- | --- | --- | --- | --- | --- | --- | --- | --- |
| Valid | 36 | 36 | 36 | 36 | 36 | 36 | 36 | 36 | 36 | 36 | 36 | 36 | 36 | 36 | 36 |
| Missing | 0 | 0 | 0 | 0 | 0 | 0 | 0 | 0 | 0 | 0 | 0 | 0 | 0 | 0 | 0 |
| Mean (arithmetic) | 1.501 | 3.197 | 4.373 | 6.484 | 9.184 | 10.55 | 13.37 | 8.847 | 8.811 | 1.415 | 4.695 | 0.812 | 3.936 | 2.487 | 9.357 |
| Std. Deviation | 1.973 | 5.302 | 4.777 | 7.163 | 11.24 | 22.68 | 16.08 | 11.14 | 10.57 | 1.916 | 7.752 | 0.855 | 3.365 | 3.278 | 15.24 |
| Shapiro-Wilk (SW) | 0.745 | 0.635 | 0.803 | 0.791 | 0.743 | 0.407 | 0.789 | 0.680 | 0.618 | 0.728 | 0.639 | 0.841 | 0.754 | 0.585 | 0.651 |
| P-value of SW | < .001 | < .001 | < .001 | < .001 | < .001 | < .001 | < .001 | < .001 | < .001 | < .001 | < .001 | < .001 | < .001 | < .001 | < .001 |
| Minimum | 0.010 | 0.053 | 0.111 | 0.542 | 0.134 | 0.131 | 0.463 | 1.186 | 1.940 | 0.000 | 0.000 | 0.007 | 0.239 | 0.232 | 0.078 |
| Maximum | 9.514 | 25.39 | 19.94 | 27.38 | 53.12 | 135.3 | 59.33 | 51.14 | 47.61 | 9.337 | 36.62 | 3.223 | 11.92 | 17.73 | 65.18 |
|  |  |  |  |  |  |  |  |  |  |  |  |  |  |  |  |

Data are presented as descriptive statistics for all gene expression variables included in the analyses shown in Figures 2–4. Normality was assessed using the Shapiro–Wilk test. For all variables, the null hypothesis of normal distribution was rejected (Shapiro–Wilk test, p < 0.001), indicating a non-normal distribution of the data. Therefore, all group comparisons were performed using the non-parametric Kruskal–Wallis test followed by Dunn’s multiple-comparison post hoc test. Variables analyzed included *Il12p40*, *Il12p35*, *Inos*, *Il10*, *Tnfα*, *Il6*, *Il1β*, *Nlrp3*, *Casp1*, *Tlr9*, *Tlr4, Tlr2*, *Ifnγ*, *Myd88*, and *Trif*.
